# Alterations of brain metrics in fetuses of women with polycystic ovary syndrome : a retrospective study based on fetal magnetic resonance imaging

**DOI:** 10.1186/s12884-021-04015-w

**Published:** 2021-08-14

**Authors:** Zhongkun Bao, Qing Zhang, Manman Pan, Xi Xi, Yuanlin Wang, Fangfang Zhang, Fangfang Wang, Yu Zou, Fan Qu

**Affiliations:** Women’s Hospital, School of Medicine, Zhejiang University, 1 Xueshi Road, Zhejiang 310006 Hangzhou, China

**Keywords:** Apparent diffusion coefficient, Brain biometry, Fetus, Magnetic resonance imaging, Polycystic ovary syndrome

## Abstract

**Background:**

Maternal polycystic ovary syndrome (PCOS) has potential detrimental effects on the neurodevelopment of offspring. This study aimed to evaluate the brain metrics in fetuses of women with PCOS based on fetal magnetic resonance imaging (MRI).

**Methods:**

This retrospective study included 60 pregnant women with PCOS (PCOS group) and 120 pregnant non-PCOS women (control group). Fetal MRI was performed followed an ultrasound and for numerous clinical indications including known or suspected fetal pathology, history of fetal abnormality in previous pregnancy or in a family member. Fetal brain biometry and apparent diffusion coefficient (ADC) value were analysed.

**Results:**

After adjusting for potential confounders, fetuses in the PCOS group showed the following characteristics compared to fetuses in the control group: (1) smaller cerebral fronto-occipital diameter (FOD), vermian height (VH) and anteroposterior diameter of the pons (APDP) (evident before 32 weeks; *P* = 0.042, *P* = 0.002 and *P* = 0.016, respectively); (2) larger left and right biparietal index (evident before 32 weeks; *P* = 0.048 and *P* = 0.025, respectively); (3) smaller left lateral ventricle (LV) (evident after 32 weeks; *P* = 0.005); (4) larger anteroposterior diameter of the vermis (APDV) and hippocampal infolding angle (HIA) (evident after 32 weeks; *P* = 0.003 and *P* < 0.001, respectively); (5) higher ADC value in frontal white matter (FWM) and in basal ganglia (BG) (evident before and after 32 weeks; all *P* < 0.05).

**Conclusions:**

There exist a different pattern of brain metrics in PCOS offspring *in utero*.

## Background

Polycystic ovary syndrome (PCOS) occurs in 5–12 % of women at reproductive age [1]. With the characteristics of hyperandrogenism, irregular menstruation and ovulatory dysfunction, PCOS accounts for 80 % of the cause of anovulatory infertility [2]. As transgenerational influences of maternal PCOS are potentially imparted to the offspring, an increasing attention is paid to the health of PCOS offspring [3]. The physical development, neurodevelopment, metabolic and reproductive profiles, endocrine status, cardiovascular feature of PCOS offspring have been presented [4]. The genetic component and the intrauterine environment of maternal PCOS, may have adverse impact on programming and developing of PCOS offspring [5]. However, it is not clear whether the impacts of maternal PCOS on offspring exist from the early development in fetal period, and few has investigated fetal characteristics of PCOS *in utero*.

Fetal magnetic resonance imaging (MRI) is a useful medical imaging modality for assessing fetal pathology, especially for fetal neurologic evaluation. Relying primarily on T2-weighted sequence, MRI examination done during pregnancy could assess the *in vivo* fetal brain maturation from 18th week of gestation to term [6]. Based on fetal and placental MRI, we have previously found that there exist alterations of fetal physical growth and development, together with decreased placental thickness in women with PCOS [7]. Considering the potential influence of maternal PCOS on neurodevelopmental health of offspring, the study hypothesized that the alteration in neurodevelopment of offspring may origin in fetal life *in utero* and the offspring of women with PCOS may show abnormal changes in brain biometry and cerebral microstructure. To verify the hypothesis, this study measured brain biometry and apparent diffusion coefficient (ADC) value in fetuses of women with PCOS by fetal MRI.

## Methods

### Study design and participants

This retrospective study included singleton pregnancies of women who attended fetal MRI and delivered with live birth infants in our hospital. The Ethics Committee approved this study with written informed consent obtained before all fetal MRI procedures. The hospital’s radiology database were queried for fetal MR imaging of totally 4132 pregnant women between 2013 and 2018. The clinical indications for fetal MRI include known or suspected fetal pathology (posterior fossa anomaly, corpus callosal anomaly, diaphragmatic hernia, microcephaly, isolated ventriculomegaly, cleft lip/palate, etc.) followed an ultrasound examination, and history of fetal abnormalities in family members or in a previous pregnancy. We excluded 2122 pregnant women because of presence of pre-existing maternal diseases (including diabetes, chronic hypertension, thyroid disease, etc.), twin or multiple pregnancies, history of maternofetal infection, abnormal fetal karyotype, any associated fetal body or brain abnormality, delivery with stillbirth, and cases in which the fetal brain was not scanned or with poor MRI image quality.

**Fig. 1:**
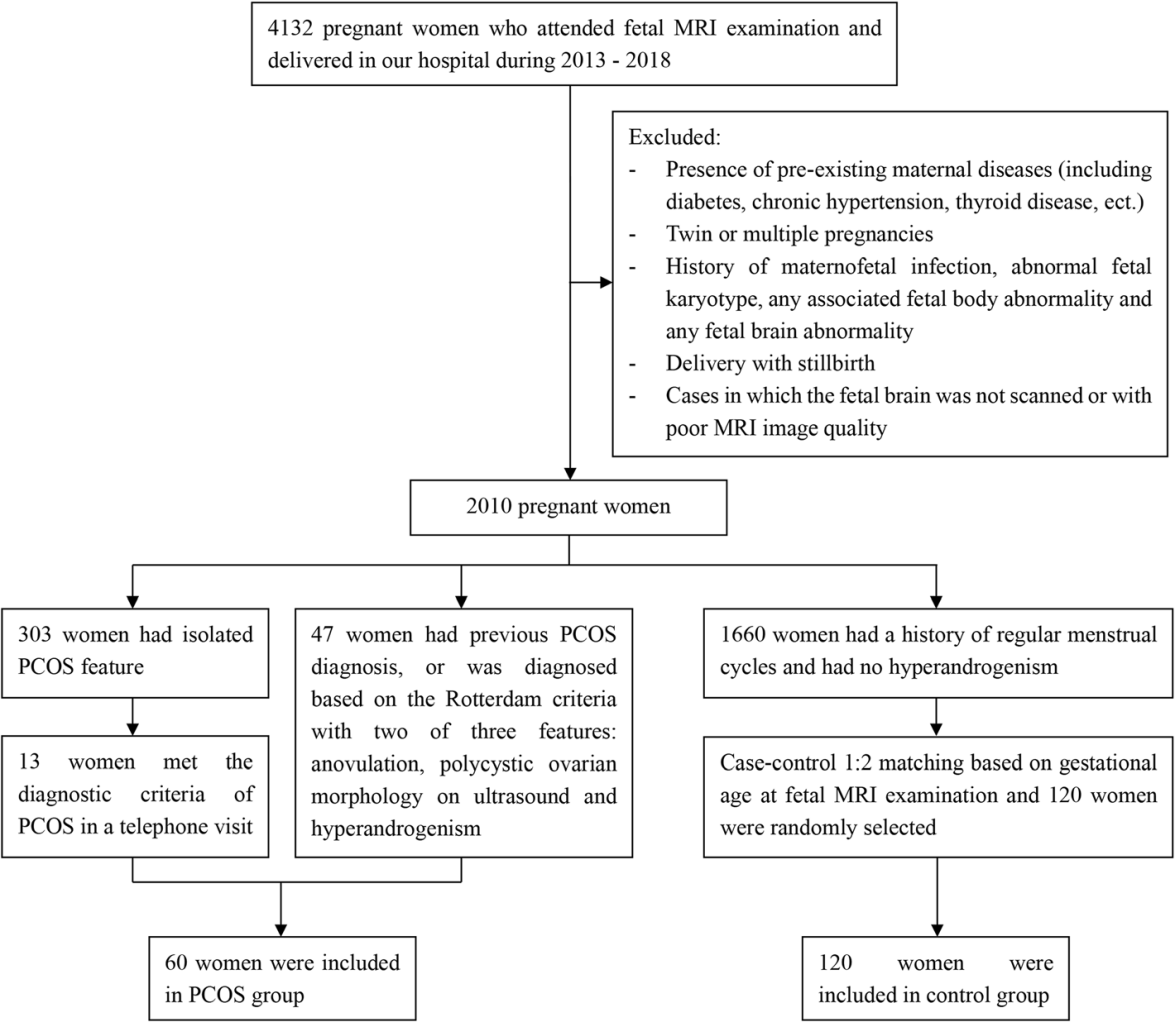
Flow chart of the sampling frame and study population. MRI, magnetic resonance imaging; PCOS, polycystic ovary syndrome

PCOS was defined according to the Rotterdam 2003 consensus criteria [8] with two of three features: anovulation, polycystic ovarian morphology on ultrasound, and hyperandrogenism. For those who had isolated PCOS feature and the information provided in database could not help to determine a PCOS diagnosis, they were telephoned and asked to participate to a survey about their gynaecological history. They would be included into PCOS group if they told they had history of previous PCOS diagnosis and provided details diagnosing PCOS in the telephone visit. As a control group, data from pregnant healthy women without PCOS were used. These women had a history of regular menstrual cycles and no clinical and/or biochemical signs of hyperandrogenism. For each women in PCOS group, two controls with a similar gestational age (GA) at the time of MRI examination were randomly selected during the same period. Finally, we identified fetal MRI examinations from 60 pregnant women with diagnosed PCOS and 120 control pregnant women. Figure 1 showed the flow chart of the sampling frame and study population.

### Magnetic resonance imaging

The fetus MRI was operated using a 1.5-Telsa (T) scanner (Signa HDxt, General Electric Company, CT, USA) with a phased- array abdominal coil. The single-shot fast spin echo (SSFSE: short repetition time/echo time (TR/TE), 3100/90 ms; bandwidth, 32 kHz; field-of-view (FOV), 30 × 32 cm; matrix, 256 × 192; slice thickness, 5 mm; gap, 0 mm; number of excitations (NEX), 1) sequence weighted images in the sagittal, axial and frontal planes were used to the maternal uterus. The single-shot fast spin echo (SSFSE: TR/TE, 3100/90 ms; bandwidth, 32 kHz; FOV, 30 × 32 cm; matrix, 256 × 192; slice thickness, 4–5 mm; gap, 0 mm; NEX, 1) and fast imaging employing steady-state acquisition (FIESTA: TR/TE, 3.6/1.7 ms; bandwidth, 80 Hz; FOV, 32 × 32 cm; matrix, 256 × 224; slice thickness, 5 mm; gap, 0 mm; flip angle, 55°) sequences on all three planes were used to the fetal brain. Diffusion-weighted imaging (DWI) of the fetal brain was performed in the axial plane with single-shot echo-planar sequence (TR/TE, 3200/88 ms; FOV, 36 cm×36 cm; slice thickness, 4 mm; b-value, 600 s/mm2). All sequences were performed with the specific absorption ratio (SAR) values lower than 2.0 W/kg.

### Data collection

**Fig. 2:**
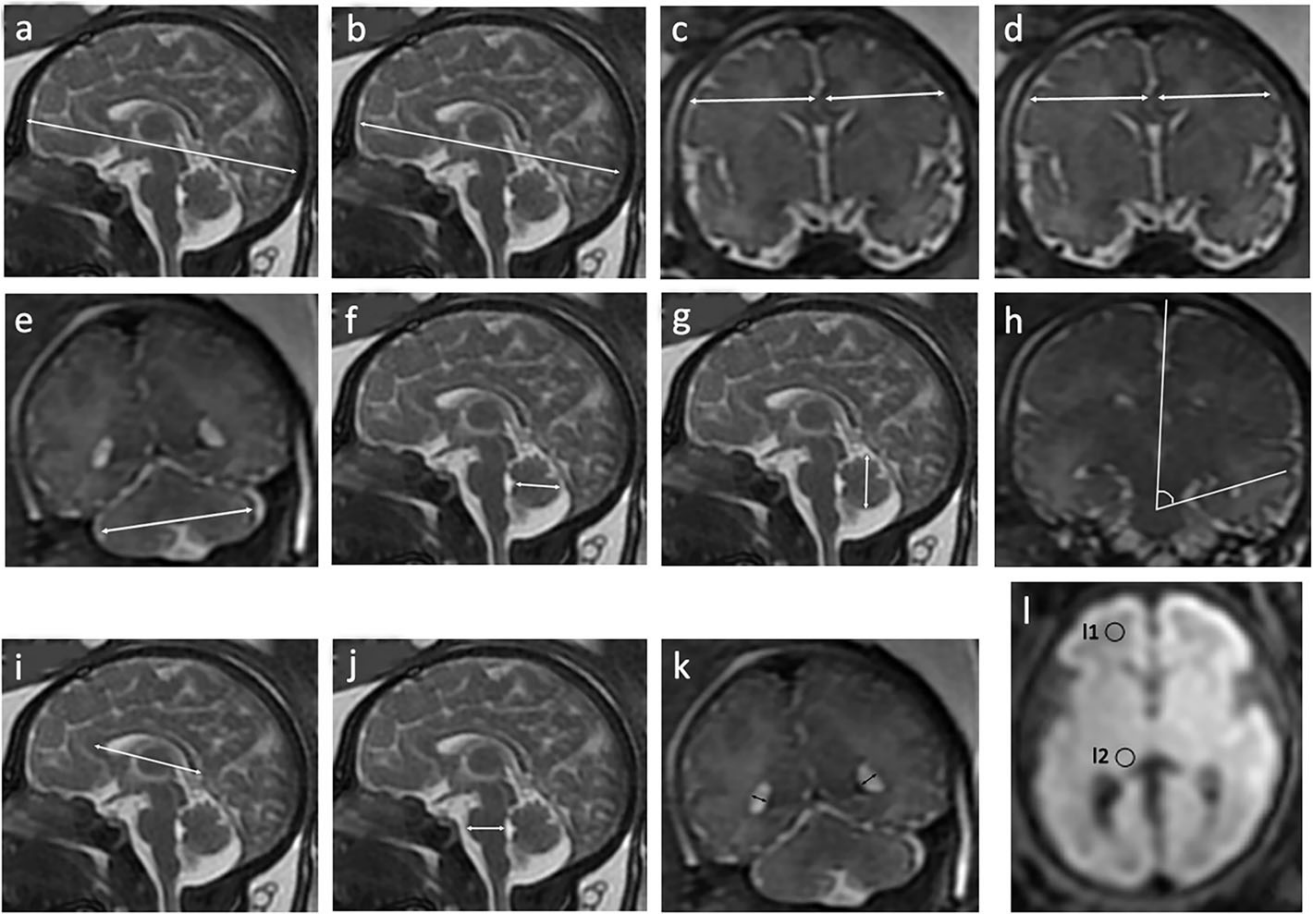
Magnetic resonance imaging slice showing measurement of: (**a**) bone fronto-occipital diameter; (**b**) cerebral fronto-occipital diameter; (**c**) left and right bone biparietal diameters; (**d**) left and right cerebral biparietal diameters; (**e**) transverse cerebellar diameter; (**f**) anteroposterior diameter of the vermis; (**g**) vermian height; (**h**) hippocampal infolding angle; (**i**) length of the corpus callosum; (**j**) anteroposterior diameter of the pons; (**k**) left and right lateral ventricles; and (**l**) apparent diffusion coefficient measures in frontal white matter (l1) and in basal ganglia (l2)

Measurements of the brain biometry and ADC value were made independently by two radiologists experienced in fetal MRI and blinded to participants’ information. The following fetal brain biometric parameters were measured: bone and cerebral fronto-occipital diameters (FODs), bone and cerebral biparietal diameters (BPDs), transverse cerebellar diameter (TCD), anteroposterior diameter of the vermis (APDV), vermian height (VH), hippocampal infolding angle (HIA), length of the corpus callosum (LCC), anteroposterior diameter of the pons (APDP) and lateral ventricles (LV). ADC values in frontal white matter (FWM) and in basal ganglia (BG) were obtained from DWI section, and ADC maps were reconstructed using the AW workstation and “Functool” software version 9.4.05a. The methodology used to measure fetal brain biometric parameters and ADC value was followed with previous studies [9–12] and detailed in Fig. 2. Maternal and infant demographic and clinical characteristics were also collected from the hospital’s electronic medical record system.

### Study size

We included data of pregnancy women with PCOS and non-PCOS controls with a similar GA at MRI examination between 2013 and 2018. Since the lack of published data on the best fetal brain MRI biometric parameters to use as primary end-point, power calculation was not feasible and the study size was determined according to the maximum number of eligible participants in consideration of the experimental design of the study.

### Statistical analysis

A Kolmogorov-Smirnov test was performed for the normality of continuous variable data. The Student’s *t* test was utilized for normally distributed quantitative data and the Mann-Whitney *U* test for non-parametric data. Categorical variables were analysed by Chi-squared test or Fisher exact tests. To analyse inter-group differences in fetal brain metrics, multivariate linear regression analysis was used with PCOS as independent and each fetal brain biometric parameter or ADC value as dependent variable. *P*-values resulting from univariate analyses were subsequently adjusted for potential confounders, including maternal age, body mass index (BMI), mode of conception, GA at MRI examination, and the presence of gestational diabetes mellitus (GDM), pre-eclampsia and preterm birth. The quadratic nonlinear analyses were used to indicate the correlation between GA and fetal brain biometric MRI parameters. For all analyses, two-sided *P*-values of < 0.05 was considered statistically significant. The SPSS version 20 (IBM, Armonk, NY, USA) was used for all statistical analyses and statistical significance was set at a level of *P* < 0.05.

## Results

### Baseline and clinical characteristics of study population

**Fig. 3:**
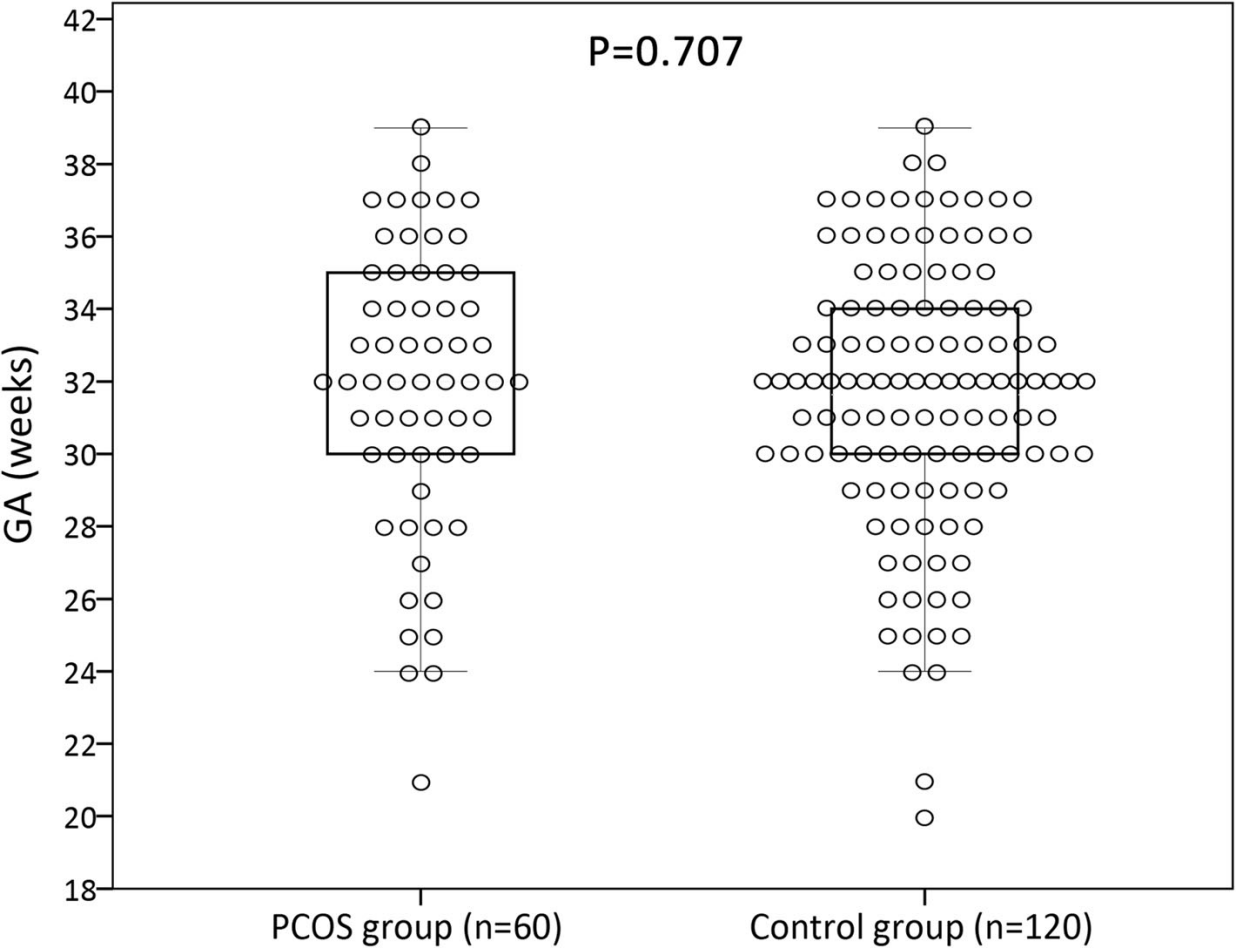
Distributions of gestational age (GA) at fetal MRI examination in polycystic ovary syndrome (PCOS) and control groups. Comparison was determined by the Mann–Whitney *U* test

As showed in Fig. 3, the median GA at fetal MRI examination was 32 weeks for both groups (interquartile range: 30, 35 in PCOS group and 30, 34 in control group), without a significant difference in distribution between the two groups (*P* = 0.707). Therefore, in the subsequent comparative analysis, sub-groups were stratified into GA ≤ 32 weeks and GA > 32 weeks. There was no significant difference in maternal demographic data between PCOS and control groups except the mode of conception (*P* < 0.001). The rate of preterm birth (< 37 weeks) in PCOS group were significantly higher than that of the controls (*P* = 0.002). The details of baseline characteristics and clinical data were shown in Table 1.

**Table 1:** Baseline and clinical characteristics

**Items**	**PCOS group **(*n*=60)	**Control group **(*n*=120)	**PCOS vs. Control** ***P*value**
**Mothers**			
Age (years)	30.6±4.0	30.3±4.0	0.666
Pre-pregnancy BMI (kg/m^2^)			
BMI<25	53 (88.3)	112 (93.3)	0.264
BMI≥25	7 (11.7)	8 (6.7)	
GA at MRI examination (weeks)			
≤32 weeks	33 (55.0)	73 (60.8)	0.453
>32 weeks	27 (45.0)	62 (39.2)	
Current employment status [n(%)]			
Full-time employment	49 (81.7)	102 (85.0)	0.562
Part-time employment	5 (8.3)	9 (7.5)	
Registered unemployed	6 (10.0)	7 (5.8)	
Others	0 (0.0)	2 (1.7)	
Educational level [n(%)]			
Less than college education	16 (26.7)	28 (23.3)	0.710
College education	38 (63.3)	83 (69.2)	
Higher than college education	6 (10.0)	9 (7.5)	
Gravidity (times)	2 (1, 2)	2 (1, 3)	0.390
Parity [n(%)]			
Nulliparity	40 (66.7)	74 (61.7)	0.512
Multiparity	20 (33.3)	46 (38.3)	
Mode of conception [n (%)]			
Nature conception	47 (78.3)	116 (96.7)	0.000
Pharmacological ovulation induction	4 (6.7)	0 (0.0)	
In-vitro fertilization	9 (15.0)	4 (3.3)	
GDM [n (%)]	14 (23.3)	15 (12.5)	0.062
Pre-eclampsia [n (%)]	5 (8.3)	3 (2.5)	0.073
PIH [n (%)]	3 (5.0)	5 (4.2)	0.798
ICP [n (%)]	3 (5.0)	3 (2.5)	0.402
Mode of delivery [n (%)]			
Vaginal delivery	28 (46.7)	69 (57.5)	0.169
Caesarean delivery	32 (53.3)	51 (42.5)	
**Offspring**			
Ultrasound biparietal diameter at MRI examination (cm)	8.09±0.86	8.18±0.97	0.566
Ultrasound femur length at MRI examination (cm)	5.92±0.78	5.99±0.83	0.574
GA at birth (weeks)	38.3±2.2	38.8±1.5	0.167
FGR	1 (1.7)	3 (2.5)	0.721
Preterm birth (<34 weeks)	3 (5.0)	1 (0.8)	0.074
Preterm birth (<37 weeks)	10 (16.7)	4 (3.3)	0.002
Gender [n (%)]			
Male	33 (55.0)	73 (60.8)	0.453
Female	27 (45.0)	47 (39.2)	
Birth weight (g)	3258.3±658.5	3300.5±563.9	0.656
Birth height (cm)	49.0±3.6	49.7±2.5	0.216
Birthweight <10th centile (SGA)	2 (3.3)	5 (4.2)	0.785
Birthweight >90th centile (LGA)	4 (6.7)	6 (5.0)	0.645
1min Apgar score [n (%)]			
<7	1 (1.7)	2 (1.7)	1.000
≥7	59 (98.3)	118 (98.3)	
5min Apgar score [n (%)]			
<7	0 (0.0)	1 (0.8)	1.000
≥7	60 (100.0)	119 (99.2)	
NICU admission [n (%)]	28 (46.7)	41 (34.2)	0.104

Note: Data was presented as mean±standard deviation (SD), median (25^th^, 75^th^ quartiles) or number (percentage). Comparisons were determined by the Student’s t-test and the chi-squared test. *PCOS* polycystic ovary syndrome, *BMI* body mass index, *GA* gestational age, *MRI* magnetic resonance imaging, *GDM* gestational diabetes mellitus, *PIH* pregnancy-induced hypertension, *ICP* intrahepatic cholestasis of pregnancy, *FGR* fetal growth restriction, *SGA* small-for-gestational age, *LGA* large-for-gestational age, *NICU* neonatal intensive care unit

### Fetal brain biometric MRI parameters and the correlation with GA

As shown in Table 2, fetal brain biometric MRI parameters in PCOS group when GA ≤ 32 weeks and > 32 weeks were compared with the control group. And Table 3 showed the results of univariate analysis and subsequently multivariate linear regression analysis to adjust for potential confounders. When GA ≤ 32 weeks, fetal VH and APDP in PCOS group was significantly smaller than control group (*P* = 0.018, 0.027, respectively). After adjustment for potential confounding variables, cerebral FOD, VH and APDP of fetuses in PCOS group were significantly smaller than control group (*P* = 0.042, 0.002, 0.016, respectively), while left and right biparietal index in PCOS group were significantly larger than control group (*P* = 0.048, 0.025, respectively). When GA > 32 weeks, left bone BPD and left cerebral BPD of fetuses in PCOS group were significantly smaller than control group (*P* = 0.034, 0.036, respectively), however, after adjusting potential confounders these differences were not statistically significant. Fetuses in PCOS group had significantly smaller left LV (*P* = 0.001) and significantly larger APDV and HIA (*P* = 0.006, 0.000, respectively) than control group, and these were still significant after adjusting potential confounders (*P* = 0.005, 0.003, 0.000, respectively).

**Table 2:** Comparisons of fetal brain biometric MRI parameters between PCOS group and control group

Parameters	PCOS group (*n*=60)		Control group (*n*=120)		PCOS vs. Control *P*-value	
	GA ≤32 weeks	GA >32 weeks	GA ≤32 weeks	GA >32 weeks	GA ≤32 weeks	GA >32 weeks
Bone FOD (mm)	90.5±11.3	102.1±6.0	94.5±9.2	104.4±6.6	0.082	0.140
Cerebral FOD (mm)	85.0±11.0	97.8±6.2	89.5±9.6	100.2±7.0	0.050	0.140
Fronto-occipital index	0.06±0.02	0.04±0.01	0.05±0.02	0.04±0.02	0.104	0.550
Left bone BPD (mm)	35.5±5.7	39.7±3.0	36.5±4.2	41.5±3.5	0.386	0.034
Left cerebral BPD (mm)	31.9±5.0	37.5±3.0	33.2±4.3	38.9±3.0	0.176	0.036
Left biparietal index	0.10±0.05	0.06±0.02	0.09±0.04	0.06±0.03	0.313	0.902
Right bone BPD (mm)	35.0±5.1	39.9±2.5	36.1±4.4	41.3±3.5	0.258	0.070
Right cerebral BPD (mm)	31.6±4.7	37.8±2.7	33.2±4.5	38.6±3.2	0.113	0.223
Right biparietal index	0.10±0.04	0.06±0.02	0.08±0.04	0.06±0.03	0.096	0.143
TCD (mm)	35.5±6.4	45.9±4.6	37.4±5.6	46.7±3.9	0.151	0.427
VH (mm)	15.8±2.6	19.1±2.3	17.2±2.9	20.1±2.4	0.018	0.081
APDV (mm)	13.0±2.2	16.4±2.4	12.6±2.2	15.0±1.7	0.395	0.006
LCC (mm)	38.4±5.9	41.3±4.5	39.1±4.8	42.2±3.7	0.516	0.391
APDP (mm)	10.5±1.9	13.3±1.3	11.3±1.6	13.4±1.1	0.027	0.630
Left LV (mm)	8.2±3.0	6.2±2.0	7.7±2.4	7.9±2.1	0.387	0.001
Right LV (mm)	7.7±2.5	6.5±2.1	7.3±2.4	7.4±2.5	0.472	0.112
HIA (°)	71.6±5.0	75.2±4.5	69.9±4.1	71.6±3.4	0.076	0.000

Note: Data was presented as mean±standard deviation (SD). Comparisons were determined by Student’s t-test. *PCOS* polycystic ovary syndrome, *GA* gestational age, *FOD* fronto-occipital diameter, *BPD* biparietal diameter, *TCD* transverse cerebellar diameter, *VH* vermian height, *APDV* anteroposterior diameter of the vermis, *LCC* length of the corpus callosum, *APDP* anteroposterior diameter of the pons, *LV* lateral ventricle, *HIA* hippocampal infolding angle, *BMI* body mass index, *MRI* magnetic resonance imaging, *GDM* gestational diabetes mellitus

**Table 3:** Association between maternal PCOS and fetal brain biometric MRI parameters

	GA ≤32 weeks		GA >32 weeks		GA ≤32 weeks		GA >32 weeks	
	β (95% CI)^a^	*P*-value^a^	β (95% CI)^a^	*P*-value^a^	β (95% CI)^b^	*P*-value^b^	β (95% CI)^b^	*P*-value^b^
Bone FOD (mm)	0.19 (-0.14, 8.12)	0.058	0.17 (-0.77, 5.38)	0.140	0.07 (-0.41, 3.32)	0.125	0.17 (-0.27, 4.77)	0.079
Cerebral FOD (mm)	0.20 (0.26, 8.63)	0.038	0.17 (-0.81, 5.63)	0.140	0.09 (0.08, 4.05)	0.042	0.18 (-0.13, 5.09)	0.062
Fronto-occipital index	-0.20 (-0.02, 0)	0.039	-0.07 (-0.01, 0.01)	0.550	-0.20 (-0.02, 0)	0.055	-0.11 (-0.01, 0)	0.355
Left bone BPD (mm)	0.10 (-0.99, 2.92)	0.328	0.25 (0.14, 3.37)	0.034	-0.05 (-1.71, 0.62)	0.354	0.21 (-0.06, 3.0)	0.059
Left cerebral BPD (mm)	0.13 (-0.59, 3.19)	0.176	0.24 (0.11, 3.17)	0.036	0.03 (-0.77, 1.44)	0.547	0.21 (-0.03, 2.80)	0.055
Left biparietal index	-0.10 (-0.03, 0.01)	0.313	-0.02 (-0.01, 0.01)	0.902	-0.21 (-0.04, 0)	0.048	-0.02 (-0.01, 0.01)	0.865
Right bone BPD (mm)	0.11 (-0.82, 3.03)	0.258	0.21 (-0.12, 2.92)	0.070	0.00 (-0.99, 1.04)	0.960	0.18 (-0.19, 2.62)	0.089
Right cerebral BPD (mm)	0.16 (-0.37, 3.43)	0.113	0.14 (-0.57, 2.42)	0.223	0.08 (-0.26, 1.85)	0.140	0.12 (-0.58, 2.13)	0.257
Right biparietal index	-0.16 (-0.03, 0)	0.096	0.17 (0, 0.02)	0.143	-0.25 (-0.04, 0)	0.025	0.16 (0, 0.02)	0.181
TCD (mm)	0.15 (-0.56, 4.39)	0.129	0.09 (-1.20, 2.81)	0.427	0.08 (-0.31, 2.42)	0.127	0.11 (-0.66, 2.45)	0.253
VH (mm)	0.22 (0.21, 2.57)	0.021	0.20 (-0.13, 2.15)	0.081	0.22 (0.51, 2.20)	0.002	0.17 (-0.23, 1.96)	0.121
APDV (mm)	-0.08 (-1.31, 0.52)	0.395	-0.32 (-2.43, -0.42)	0.006	-0.15 (-1.40, 0.03)	0.060	-0.34 (-2.50, -0.54)	0.003
LCC (mm)	0.06 (-1.44, 2.84)	0.516	0.10 (-1.09, 2.74)	0.391	-0.07 (-2.22, 0.66)	0.281	0.06 (-1.41, 2.36)	0.617
APDP (mm)	0.22 (0.09, 1.49)	0.027	0.06 (-0.43, 0.71)	0.630	0.14 (0.10, 0.89)	0.016	0.04 (-0.40, 0.60)	0.695
Left LV (mm)	-0.09 (-1.57, 0.62)	0.387	0.37 (0.70, 2.66)	0.001	-0.16 (-2.17, 0.35)	0.156	0.32 (0.46, 2.44)	0.005
Right LV (mm)	-0.07 (-1.4, 0.65)	0.472	0.19 (-0.22, 2.02)	0.112	-0.14 (-1.88, 0.45)	0.228	0.19 (-0.25, 2.11)	0.120
HIA (°)	-0.17 (-3.50, 0.18)	0.076	-0.41 (-5.41, -1.71)	0.000	-0.16 (-3.63, 0.65)	0.170	-0.44 (-5.67, -1.80)	0.000

Note: Data presented as β-coefficients (95% confidence interval). β represents the increase or decrease in parameters in the PCOS group as compared with the control group. ^a^Statistical testing performed by univariate linear regression analysis. ^b^Statistical testing performed by multivariate linear regression analysis. *P*-value adjusted for maternal age, BMI, mode of conception, GA at MRI examination, and the presence of GDM, pre-eclampsia and preterm birth (<37 weeks).*PCOS* polycystic ovary syndrome, *GA* gestational age, *CI* confidence interval, *FOD* fronto-occipital diameter, *BPD* biparietal diameter, *TCD* transverse cerebellar diameter, *VH* vermian height, *APDV* anteroposterior diameter of the vermis, *LCC* length of the corpus callosum, *APDP* anteroposterior diameter of the pons, *LV* lateral ventricle, *HIA* hippocampal infolding angle, *BMI* body mass index, *MRI* magnetic resonance imaging, *GDM* gestational diabetes mellitus

**Fig. 4:**
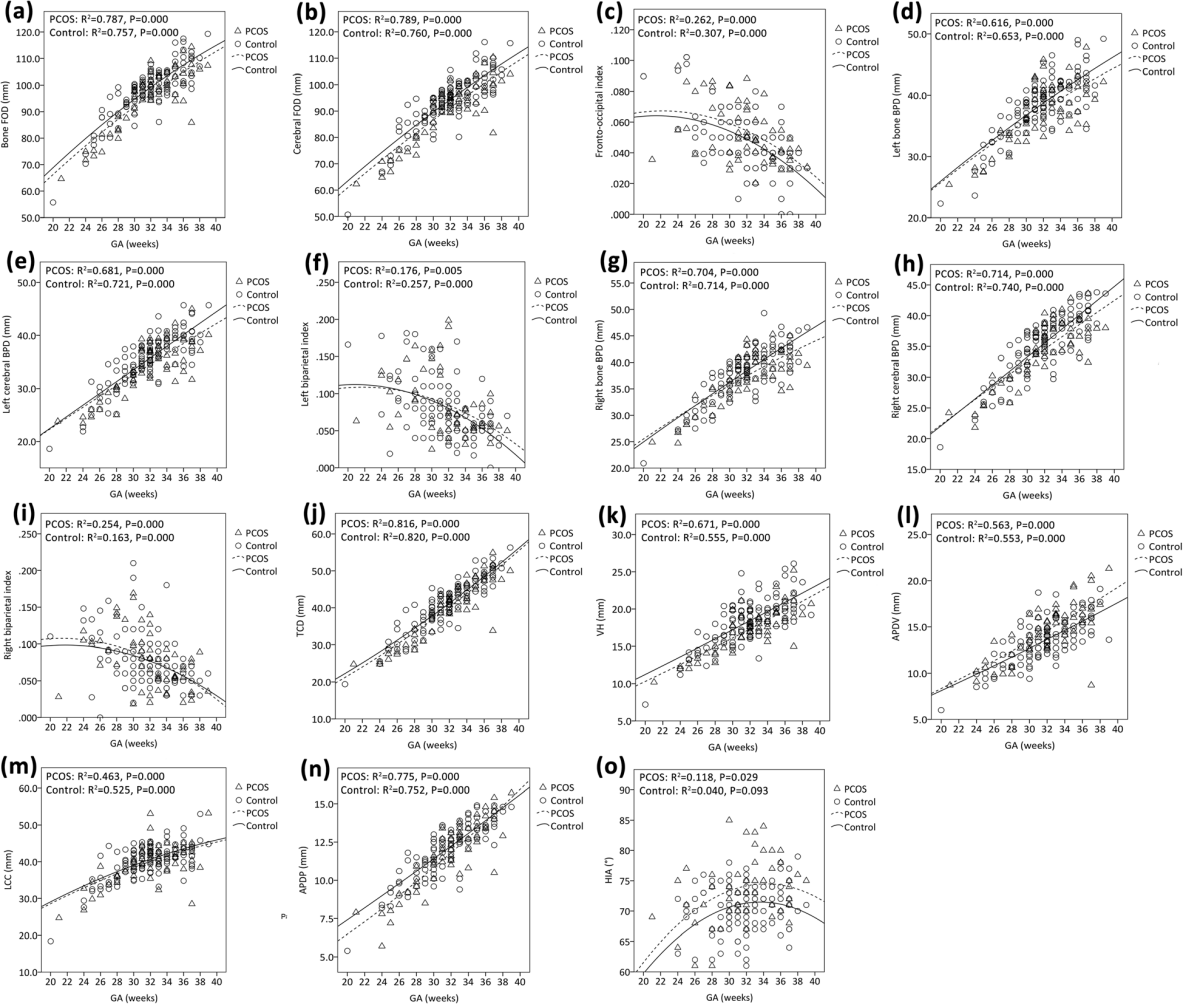
Regression curves of metrics of brain size versus gestational age (GA) in polycystic ovary syndrome (PCOS) group (n = 60) and control group (n = 120). **a** Bone fronto-occipital diameters (FOD); **b** cerebral FOD; **c** fronto-occipital index; **d** left bone biparietal diameter (BPD); **e** left cerebral BPD; **f** left biparietal index; **g** right bone BPD; **h** right cerebral BPD; **i** right biparietal index; **j** transverse cerebellar diameter (TCD); **k** vermian height (VH); **l** anteroposterior diameter of the vermis (APDV); **m** length of the corpus callosum (LCC); **n** anteroposterior diameter of the pons (APDP); **o** hippocampal infolding angle (HIA)

For the GA range of 20–39 weeks, a nonlinear quadratic model was the best fit for the change values of all the biometric parameters except left and right LV. Quadratic regression analyses suggested a significant association between GA and these parameters in both groups (all *P* < 0.01) (Fig. 4).

### ADC value and the correlation with GA

**Fig. 5:**
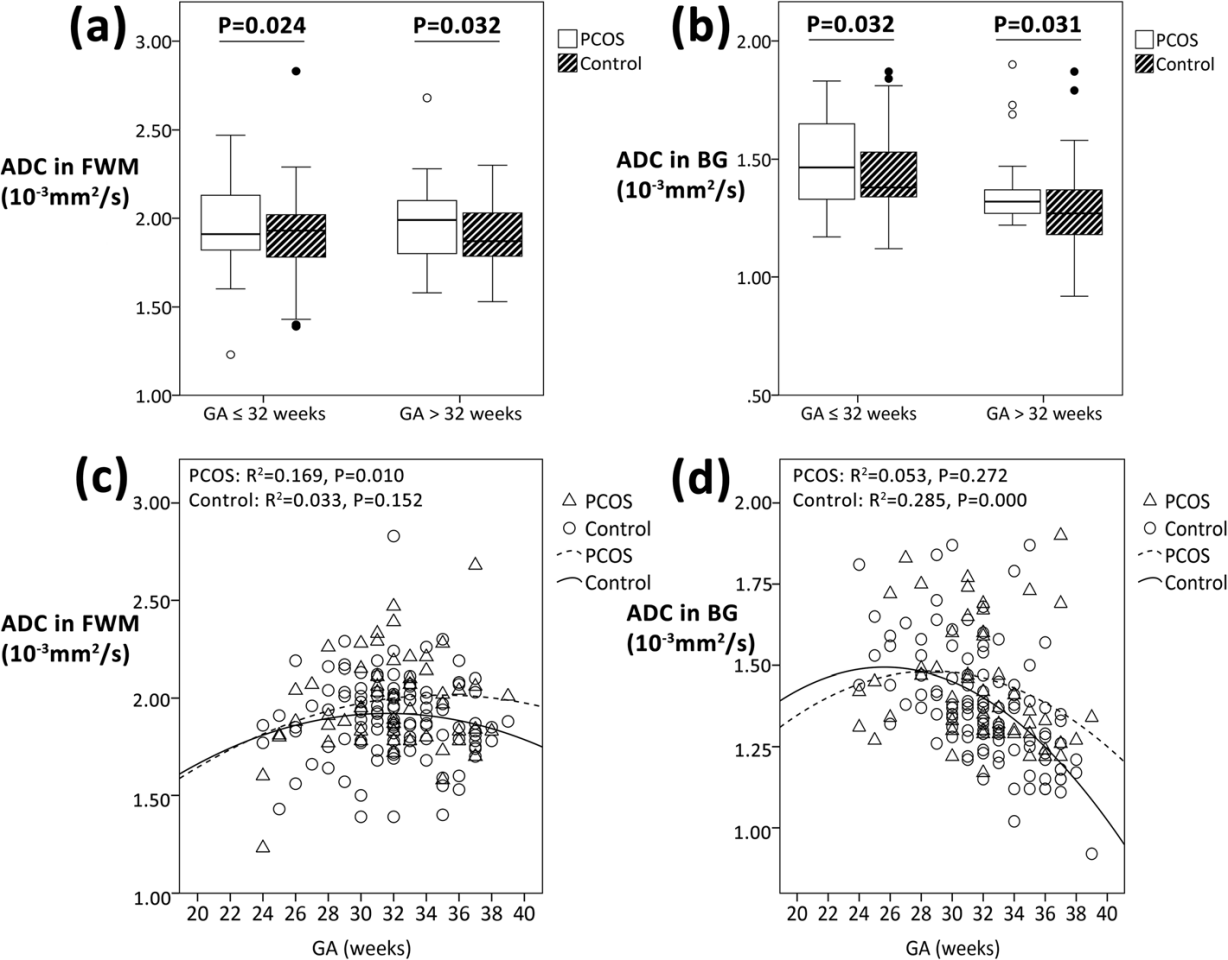
Comparisons of ADC value in FWM (**a**) and in BG (**b**) between PCOS group and control group when GA ≤ 32 weeks (PCOS: n = 31; control: n = 69) and > 32 weeks (PCOS: n = 22; control: n = 47), *P*-value adjusted for maternal age, BMI, mode of conception, GA at MRI examination, and the presence of GDM, pre-eclampsia and preterm birth (< 37 weeks). And regression curves of ADC value in FWM (**c**) and in BG (**d**) versus GA in PCOS group (n = 53) and control group (n = 116). ADC, apparent diffusion coefficient; FWM, frontal white matter; BG, basal ganglia; PCOS, polycystic ovary syndrome; GA, gestational age; BMI, body mass index; MRI, magnetic resonance imaging; GDM, gestational diabetes mellitus

There were 53 in PCOS group and 116 in control group with DWI available among the overall fetal MRI examinations. After adjusting for potential confounders, ADC value in FWM and in BG in PCOS group was significantly higher than the control group both when GA ≤ 32 weeks (*P* = 0.024, 0.032, respectively) and when GA > 32 weeks (*P* = 0.032, 0.031, respectively) (Fig. 5a-b). Quadratic regression analyses suggested a significant association between GA and ADC value of FWM in PCOS group (*P* = 0.010), and also between GA and ADC value of BG in the control group (*P* < 0.001) (Fig. 5c-d).

## Discussion

The current study assessed the pattern of fetal brain growth *in utero* in offspring of women with PCOS by measuring brain MRI biometry and ADC value. Fetuses of women with PCOS appeared to show a smaller cerebral size and a larger peri-cerebral space, which was reflected by MRI measurements of smaller cerebral FOD and higher biparietal index than fetuses of women without PCOS. All these inter-group differences have been adjusted for confounding factors including baseline characteristics and pregnancy complications. Moreover, fetuses of women with PCOS have alteration in morphometry of cerebral structures, indicated by smaller VH, APDP and left LV, larger APDV and HIA, and elevated ADC values in FWM and in BG.

Fetal brain biometric MRI parameters has become an approach to observe fetal growth [10]. Brain metrics at term-equivalent age displayed shorter bifrontal and biparietal diameters and TCD in preterm infants in contrast to full term infants [13, 14]. In fetuses of fetal growth restriction (FGR), the total brain, cerebral and cerebellar volumes were smaller compared to control fetuses [15]. Fetal BPD after 24 weeks’ GA could be detected as a predictor of small-for-gestational age (SGA)[16]. Although he prevalence of SGA and FGR in our study was not different between groups, the small cerebral size and increased peri-cerebral space in fetuses of women with PCOS suggest a delayed brain growth and might be signs of FGR or SGA, or more prone to develop preterm birth, which support those studies reporting that new-borns of women with PCOS were more frequently to be delivered with SGA [17]. In accordance with the present result, a previous study has found that fetuses of women with PCOS exhibited smaller BPD at 32 weeks of GA and shorter body length at birth, indicating maternal PCOS has a potential restrictive effect on fetal growth and development [18].

The cerebellum plays an important part in motor tasks and numerous higher functions such as learning, memory, attention, cognition, and behaviour [19]. We found an abnormal cerebellar morphometry in fetuses of women with PCOS, as indicated by smaller VH and larger APDV of cerebellum. Although we failed to get the growth pattern of cerebellum in fetuses of women with PCOS, the opposite trend of alterations in VH and APDV of cerebellum may be associated with the change in cerebellar functions, as the different growth rates of cerebellar subregions are related to their functional characteristics [20]. Furthermore, fetuses of women with PCOS presented increased HIA value after 32 gestational weeks, reflecting an abnormal hippocampal rotation process associated with hippocampal development or other cortical malformations [11]. However, it is unclear whether the altered cerebellar morphometry and HIA value, together with the decreased width of left LV in fetuses of women with PCOS found in the present study, is associated with the neurodevelopmental outcomes in postnatal life, which deserves further research.

ADC value decrease during fetal development, which is a quantitative approach to assess the morphologic brain maturity *in utero*. The increased ADC value in fetal brain is associated with aberrant signal intensity in white matter which may indicate brain histologic injuries such as the presence of vasogenic edema and astrogliosis [21]. The BG is anatomically near to the lateral ventricles and ADC value in BG is theoretically more prone to be influenced by a raised pressure in the ventricle[22]. However, this theory does not explain the decreased width of left LV in fetuses of women with PCOS found in the present study. Thus, we assumed that mechanisms other than intraventricular pressure cause elevation of the ADC in the BG.

The implications of different pattern of fetal brain metrics for later neurocognitive development are not yet clear. However, previous studies found that brain metrics of preterm new-borns could predict their developmental level of cognitive and motor function at 2 years of corrected age [14]. The alterations in morphometry of cerebellum and brain stem in SGA fetuses were correlated with neurocognitive and neurobehavior during the neonatal phase [23]. Thus, the different brain biometric MRI parameters in fetuses of women with PCOS compared to fetuses of non-PCOS women in this study, may provide the hint to the neurodevelopmental disorders that occur in postnatal life. As several population-based studies have reported, maternal PCOS may have broader detrimental impacts on neurodevelopment of the offspring, resulting in increased risk for developmental delay [24], pervasive developmental disorders (PPDs)[25], autism spectrum disorders (ASD) and attention-deficit/hyperactivity disorder (ADHD)[26].

Although the present study provides the first biometric MRI evidence concerning alterations of *in utero* brain metrics of fetuses of PCOS women, the conclusion appears limited because of the small sample size and the retrospective study design. Lack of 3D reconstruction of fetal brain makes it failed to conduct super resolution-based volumetric measurements. And the association of PCOS phenotype and the presence of fetal brain abnormalities needs further investigation. Limitations of the study also include the absence of unmeasurable images for some of the cases and the lack of long-term follow-up data for neurodevelopment outcomes. Nevertheless, the implication of our findings is ensured by the robust design, high-quality fetal MRI measurements and comprehensive data collection. In addition, as maternal prenatal characteristics and pregnancy complications were reported to be associated with human fetal brain development [27–30], in this study all results were adjusted for various potential confounders such as maternal age, prenatal BMI, mode of conception, and the presence of GDM, pre-eclampsia and preterm birth, making the cohorts comparable.

## Conclusions

In conclusion, the present study found a different pattern of brain metrics in PCOS offspring *in utero* measured by fetal MRI.

## Data Availability

The datasets analysed during the current study are available from the corresponding author on reasonable request.

## Abbreviations

PCOS: Polycystic ovary syndrome
MRI: Magnetic resonance imaging
ADC: Apparent diffusion coefficient
GA: Gestational age
DWI: Diffusion-weighted imaging
FOD: Fronto-occipital diameter
BPD: Biparietal diameter
TCD: Transverse cerebellar diameter
APDV: Anteroposterior diameter of the vermis
VH: Vermian height
HIA: Hippocampal infolding angle
LCC: Length of the corpus callosum
APDP: Anteroposterior diameter of the pons
LV: Lateral ventricles
FWM: Frontal white matter
BG: Basal ganglia
BMI: Body mass index
GDM: Gestational diabetes mellitus
SD: Standard deviation
PIH: Pregnancy-induced hypertension
ICP: Intrahepatic cholestasis of pregnancy
NICU: Neonatal intensive care unit
FGR: Fetal growth restriction
SGA: Small-for-gestational age
LGA: Large-for-gestational age
PPD: Pervasive developmental disorder
ASD: Autism spectrum disorder
ADHD: Attention-deficit/hyperactivity disorder
